# Crystal structure of 5-{3-[2,6-dimethyl-4-(5-methyl-1,2,4-oxa­diazol-3-yl)phen­oxy]prop­yl}-*N*-(11-hy­droxy­undec­yl)isoxazole-3-carboxamide hemihydrate

**DOI:** 10.1107/S2056989015007367

**Published:** 2015-04-18

**Authors:** K. Salorinne, T. Lahtinen

**Affiliations:** a Department of Chemistry, Nanoscience Center, University of Jyväskylä, PO Box 35, 40014 JYU, Finland

**Keywords:** crystal structure, anti­viral, WIN derivative, isoxazole, oxa­diazole

## Abstract

The title compound, C_29_H_42_N_4_O_5_·0.5H_2_O, comprises four structural units. A flexible prop­yloxy unit in a *gauche* conformation, with a –C(H_2_)—C(H_2_)—C(H_2_)—O– torsion angle of −64.32 (18)°, connects an isoxazole ring and an approximately planar phenyl­oxa­diazole ring system [with a maxixmum devation of 0.061 (2) Å], which are oriented almost parallel to one another with a dihedral angle of 10.75 (7)°. Furthermore, a C_11_-alkyl chain with a terminal hy­droxy group links to the 3-position of the isoxazole ring *via* an amide bond. In the crystal, a half-occupancy solvent water mol­ecule connects to a neighbouring mol­ecule *via* an inter­molecular O—H⋯O(water) hydrogen bond to the C_11_-alkyl chain hy­droxy group.

## Abstract 2

The crystal structure and supra­molecular features of 5-{3-[2,6-dimethyl-4-(5-methyl-1,2,4-oxa­diazol-3-yl)phen­oxy]prop­yl}-*N*-(11-hy­droxy­undec­yl)isoxazole-3-carboxamide hemihydrate, a derivative of anti­viral ‘WIN compounds’, are reported.

## Chemical context   

An anti­viral drug family of the so-called ‘WIN compounds’ was developed against various human illnesses caused by enteroviruses including common respiratory infections, rash or mild fever and serious or life-threatening infections, such as meningitis, myocarditis, encephalitis and paralytic poliomyelitis (De Palma *et al.*, 2008 ▸; Diana, 2003 ▸). The WIN compounds were particularly designed to target the early events (attachment, entry and uncoating) of viral replication and they have been shown to bind specifically into the inter­ior hydro­phobic pocket located at the VP1 protein of the enterovirus capsid and replacing the naturally occurring myristic acid (Reisdorph *et al.*, 2003 ▸; Giranda *et al.*, 1995 ▸; Zhang *et al.*, 2004 ▸; Thibaut *et al.*, 2012 ▸). The anti­viral drug candidate development finally led to the WIN 63843 analogue, better known as Pleconaril, which showed a drastic decrease in the metabolic degradation of the mol­ecule and a broad range of anti­viral activity against enteroviruses (Pevear *et al.*, 1999 ▸; Wildenbeest *et al.*, 2012 ▸). The design of the title compound is based on the chemical structure of the WIN 61893 analogue (Diana *et al.*, 1995 ▸), to which an additional C_11_-alkyl linker arm having a hy­droxy end group was attached at the 3-position of the isoxazole ring *via* an amide bond.
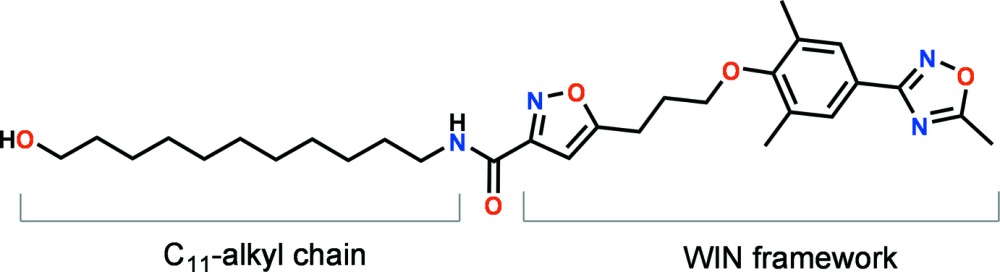


## Structural commentary   

**Figure 1:**
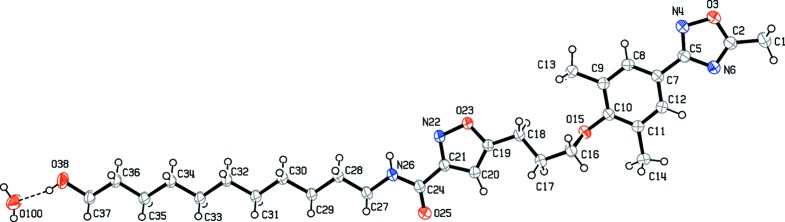
The mol­ecular structure of the title compound with the atom labelling. Displacement ellipsoids are drawn at the 50% probability level.

The mol­ecular structure of the title compound is shown in Fig. 1 ▸. The structure contains three essentially planar heterocyclic or aromatic rings, *i.e.* isoxazole (atoms C19–C21/N22/O23), benzene (C7–C12) and oxa­diazole (C2/O3/N4/C5/N6), of which the latter two are directly connected *via* atoms C7 and C5. The three heterocyclic rings are approximately coplanar to one another, having dihedral angles between the rings of 11.57 (8) (C19–C21/N22/O23 and C7–C12), 10.68 (9) (C19–C21/N22/O23 and C2/O3/N4/C5/N6) and 4.81 (9)° (C7–C12 and C2/O3/N4/C5/N6), maintaining the WIN framework in a linear conformation. The dihedral angle between the isoxazole ring (C19–C21/N22/O23) and the approximately planar phenyl­oxa­diazole ring system [C7–C12/C2/O3/N4/C5/N6, with a maximum devation of 0.061 (2)Å for atom C12] is 10.75 (7)°. The isoxazole and phenyl­oxa­diazole ring systems are connected by a prop­yloxy unit (O15–C18), which is in a *gauche* conformation, with a C18—C17—C16—O15 torsion angle of −64.32 (18)°. The amide group (N26–C24) at the 3-position of the isoxazole ring which joins the C_11_-alkyl chain (C27–O38) and the WIN framework is likewise almost coplanar with the isoxazole ring, with a dihedral angle of 10.92 (9)° between the amide (H26/N26/C24/O25) and isoxazole planes. The amide hydrogen (H26) and the acidic isoxazole hydrogen (H20) are on opposite sides, with a torsion angle (N26—C24—C21—C20) of 172.31 (15)°. The C_11_-alkyl chain (C27–C37) is in an all-*anti* conformation, with an average torsion angle of 178.80°. The WIN framework and the C_11_-linker arm structural units are aligned roughly in a 160° angle and the total length of the title mol­ecule measures up to 3.4 nm.

## Supra­molecular features   

**Figure 2:**
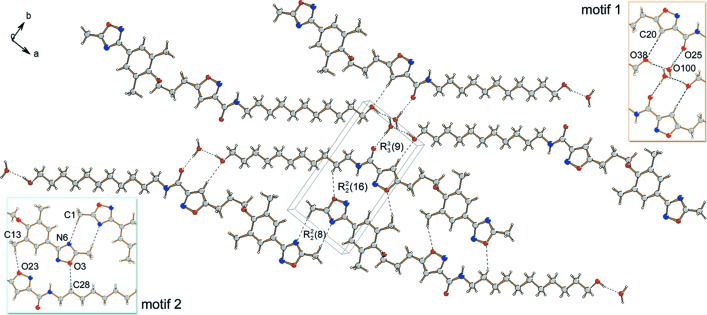
A view along the *c* axis of the crystal packing of the title compound. Inter­molecular inter­actions formed between neighbouring mol­ecules highlighting the solvent water mediated hydrogen bonding network (motif 1, orange box) and the two coordination loops between the heterocyclic isoxazole and phenyl- oxa­diazole units (motif 2, blue box).

The title compound packs in the crystal lattice in layers, in which the mol­ecules are held together by solvent-mediated O—H⋯O and C—H⋯O hydrogen bonds (motif 1), as well as C—H⋯N and C—H⋯O inter­molecular inter­actions between the heterocyclic isoxazole and phenyl­oxa­diazole units of neighbouring mol­ecules (motif 2) (Table 1 ▸). In the solvent-mediated assembly, an inter­molecular hydrogen-bonded network of the type 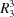
(9) is formed between the C_11_-alkyl chain hy­droxy [O—H⋯O = 1.90 (1) Å], solvent water [O—H⋯O = 1.87 (1) Å], amide carbonyl and isoxazole hydrogen (C—H⋯O = 2.56 Å) groups of two parallel neighbouring mol­ecules (Fig. 2 ▸). In a similar manner, two pairs of C—H⋯N and C—H⋯O hydrogen bonds connect three opposite-facing neighbouring mol­ecules *via R*_2_^2^(8) and 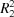
(16) loops between the isoxazole (C—H⋯O = 2.51 Å) and phenyl­oxa­diazole (C—H⋯O = 2.64 Å and C—H⋯N = 2.65 Å) groups (Fig. 2 ▸).

## Database survey   

A search of the Cambridge Structural Database (CSD; Version 5.36, November 2014; Groom & Allen, 2014 ▸) revealed the presence of nine structures (CSD refcode VOGDAY contains two independent mol­ecules; Salorinne *et al.*, 2014 ▸) with the substructure 3-{3,5-dimethyl-4-[3-(3-methyl­isoxazol-5-yl)prop­oxy]phen­yl}-5-methyl-1,2,4-oxa­diazole. These nine structures belong to three similar compounds of 5-{3-[2,6-dimethyl-4-(5-methyl-1,2,4-oxa­diazol-3-yl)phen­oxy]prop­yl}iso­xazole-3-carb­oxy­lic acid (Salorinne *et al.*, 2014 ▸), ethyl 5-{3-[2,6-dimethyl-4-(5-methyl-1,2,4-oxa­diazol-3-yl)phen­oxy]prop­yl}iso­xazole-3-carboxyl­ate (Salorinne *et al.*, 2014 ▸) and 3-{3,5-di­methyl-4-[3-(3-methyl­isoxazol-5-yl)prop­oxy]phen­yl}-5-tri­fluoro­methyl-1,2,4-oxa­diazole (Coste *et al.*, 2004 ▸). In six of the nine structures (CSD refcodes VOGCOL01, VOGDAY, HAJYUN, HAJYUN01, HAJYUN02 and HAJYUN03; Salorinne *et al.*, 2014 ▸; Coste *et al.*, 2004 ▸), the isoxazole and phenyl­oxa­diazole heterocyclic rings of the WIN framework are almost coplanar, similar to the title compound. However, in two of the structures (CSD refcodes VOGCOL and VOGDEL; Salorinne *et al.*, 2014 ▸), the heterocyclic ring systems are tilted slightly with angles of 34–38° between the ring planes, whereas in one of the structures (CSD refcode VOGCOL; Salorinne *et al.*, 2014 ▸), the heterocyclic ring systems are closer to a perpendicular orientation, with an angle of *ca* 60.8°. In all of the structures, the prop­yloxy unit is in a *gauche* conformation, with torsion angles in the range 62.4–69.2°.

## Synthesis and crystallization   

An amide coupling reaction of 5-{3-[2,6-dimethyl-4-(5-methyl-1,2,4-oxa­diazol-3-yl)phen­oxy]prop­yl}isoxazole-3-carb­oxy­lic acid (0.17 mmol, Salorinne *et al.*, 2014 ▸) with 11-amino-1-undeca­nol (0.18 mmol) in di­chloro­methane (20 ml) in the presence of *N*-[3-(di­methyl­amino)­prop­yl]-*N*-ethyl­carbodi­imide (0.19 mmol) and a catalytic amount of 1-hy­droxy­benzotriazole at 273 K gave the title compound in 68% yield after subsequent chromatographic purification in silica with a di­chloro­methane–methanol mixture (95:5 *v*/*v*). Needle-like crystals of the title compound were obtained from an ethanol solution by vapor diffusion with water.

## Refinement   

Crystal data, data collection and structure refinement details are summarized in Table 2 ▸. All H atoms were positioned geometrically and allowed to ride on their parent atoms, with C—H = 0.95–0.99 Å, and with *U*_iso_(H) = 1.5*U*_eq_(C) for methyl and 1.2*U*_eq_(C) for other H atoms, and N—H = 0.88 Å and *U*_iso_(H) = 1.2*U*_eq_(N). The positions of the O-bound H atoms were located in a difference Fourier map and refined as riding atoms with *U*_iso_(H) = 1.5*U*_eq_(O). The O—H distance of the half-occupied water molecule was restrained to 0.84 (1) Å.

## Supplementary Material

Crystal structure: contains datablock(s) I. DOI: 10.1107/S2056989015007367/lh5758sup1.cif

Structure factors: contains datablock(s) I. DOI: 10.1107/S2056989015007367/lh5758Isup2.hkl

Supporting information file. DOI: 10.1107/S2056989015007367/lh5758Isup3.cml

CCDC reference: 1059505

Additional supporting information:  crystallographic information; 3D view; checkCIF report

Enhanced figure: interactive version of Fig. 3

## Figures and Tables

**Table 1:** Hydrogen-bond geometry (, )

*D*H*A*	*D*H	H*A*	*D* *A*	*D*H*A*
C28H28*A*O3^i^	0.99	2.64	3.2567(19)	120
C20H20O38^ii^	0.95	2.56	3.505(2)	175
C13H13*B*O23^i^	0.98	2.51	3.416(2)	154
C1H1*B*N6	0.98	2.65	3.622(2)	174
O100H10*B*O25^iii^	0.84(1)	1.87(1)	2.710(3)	180(6)
O38H38O100	0.82(1)	1.90(1)	2.695(4)	164(2)

Symmetry codes: (i) 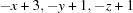; (ii) 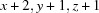; (iii) 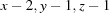.

**Table 2:** Experimental details

Crystal data	
Chemical formula	2C_29_H_42_N_4_O_5_H_2_O
*M*_r_	1071.34
Crystal system, space group	Triclinic, *P* 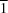
Temperature (K)	170
*a*, *b*, *c* ()	6.7137(3), 14.0263(5), 16.6757(8)
, , ()	113.889(4), 94.515(4), 90.976(4)
*V* (^3^)	1429.29(12)
*Z*	1
Radiation type	Mo *K*
(mm^1^)	0.09
Crystal size (mm)	0.42 0.15 0.09

Data collection	
Diffractometer	Agilent SuperNova, Single source at offset, Eos
Absorption correction	Analytical [*CrysAlis PRO* (Agilent, 2013 ▸), based on expressions derived by Clark Reid (1995 ▸)]
*T*_min_, *T*_max_	0.990, 0.998
No. of measured, independent and observed [*I* > 2(*I*)] reflections	13976, 7670, 5463
*R*_int_	0.016
(sin /)_max_ (^1^)	0.716

Refinement	
*R*[*F* ^2^ > 2(*F* ^2^)], *wR*(*F* ^2^), *S*	0.053, 0.148, 1.05
No. of reflections	7670
No. of parameters	364
No. of restraints	3
H-atom treatment	H atoms treated by a mixture of independent and constrained refinement
_max_, _min_ (e ^3^)	0.36, 0.26

Computer programs: *CrysAlis PRO* (Agilent, 2013 ▸), *SHELXS97* (Sheldrick, 2008 ▸), *SHELXL2013* (Sheldrick, 2015 ▸) and *OLEX2* (Dolomanov *et al.*, 2009 ▸).

## supplementary crystallographic information

### Crystal data

**Table:**

2C_29_H_42_N_4_O_5_·H_2_O	*Z* = 1
*M**_r_* = 1071.34	*F*(000) = 578
Triclinic, *P*1	*D*_x_ = 1.245 Mg m^−^^3^
*a* = 6.7137 (3) Å	Mo *K*α radiation, λ = 0.71073 Å
*b* = 14.0263 (5) Å	Cell parameters from 5261 reflections
*c* = 16.6757 (8) Å	θ = 2.5–30.3°
α = 113.889 (4)°	µ = 0.09 mm^−^^1^
β = 94.515 (4)°	*T* = 170 K
γ = 90.976 (4)°	Needle, clear colourless
*V* = 1429.29 (12) Å^3^	0.42 × 0.15 × 0.09 mm

### Data collection

**Table:**

Agilent SuperNova, Single source at offset, Eos diffractometer	7670 independent reflections
Radiation source: SuperNova (Mo) X-ray Source	5463 reflections with *I* > 2σ(*I*)
Mirror monochromator	*R*_int_ = 0.016
Detector resolution: 16.0107 pixels mm^-1^	θ_max_ = 30.6°, θ_min_ = 2.5°
ω scans	*h* = −8→9
Absorption correction: analytical [*CrysAlis PRO* (Agilent, 2013), based on expressions derived by Clark & Reid (1995)]	*k* = −19→20
*T*_min_ = 0.990, *T*_max_ = 0.998	*l* = −23→23
13976 measured reflections	

### Refinement

**Table:**

Refinement on *F*^2^	Primary atom site location: structure-invariant direct methods
Least-squares matrix: full	Hydrogen site location: mixed
*R*[*F*^2^ > 2σ(*F*^2^)] = 0.053	H atoms treated by a mixture of independent and constrained refinement
*wR*(*F*^2^) = 0.148	*w* = 1/[σ^2^(*F*_o_^2^) + (0.0537*P*)^2^ + 0.3588*P*] where *P* = (*F*_o_^2^ + 2*F*_c_^2^)/3
*S* = 1.05	(Δ/σ)_max_ = 0.001
7670 reflections	Δρ_max_ = 0.36 e Å^−^^3^
364 parameters	Δρ_min_ = −0.26 e Å^−^^3^
3 restraints	

### Special details

**Table:**

Experimental. Absorption correction: [CrysAlisPro (Agilent, 2013). Analytical numeric absorption correction using a multifaceted crystal model based on expressions derived by Clark & Reid (1995)
Geometry. All e.s.d.'s (except the e.s.d. in the dihedral angle between two l.s. planes) are estimated using the full covariance matrix. The cell e.s.d.'s are taken into account individually in the estimation of e.s.d.'s in distances, angles and torsion angles; correlations between e.s.d.'s in cell parameters are only used when they are defined by crystal symmetry. An approximate (isotropic) treatment of cell e.s.d.'s is used for estimating e.s.d.'s involving l.s. planes.

### Fractional atomic coordinates and isotropic or equivalent isotropic displacement parameters (Å^2^)

**Table:**

	*x*	*y*	*z*	*U*_iso_*/*U*_eq_	Occ. (<1)
N26	0.4579 (2)	0.58797 (10)	0.29818 (9)	0.0368 (3)	
H26	0.5168	0.5282	0.2794	0.044*	
O15	1.50948 (17)	0.80964 (8)	0.69521 (7)	0.0345 (3)	
O23	0.98187 (18)	0.57258 (8)	0.43787 (8)	0.0419 (3)	
C33	−0.5511 (2)	0.30398 (12)	−0.11261 (10)	0.0345 (4)	
H33A	−0.6504	0.3164	−0.0691	0.041*	
H33B	−0.5519	0.3632	−0.1305	0.041*	
C28	0.1978 (2)	0.49429 (12)	0.17862 (10)	0.0333 (3)	
H28A	0.2024	0.4347	0.1960	0.040*	
H28B	0.2944	0.4832	0.1342	0.040*	
C27	0.2593 (2)	0.59363 (13)	0.25821 (10)	0.0367 (4)	
H27A	0.2598	0.6531	0.2406	0.044*	
H27B	0.1607	0.6061	0.3021	0.044*	
C35	−0.8237 (3)	0.20232 (12)	−0.23541 (10)	0.0364 (4)	
H35A	−0.8320	0.2614	−0.2534	0.044*	
H35B	−0.9211	0.2122	−0.1915	0.044*	
O25	0.4853 (2)	0.75915 (9)	0.38872 (8)	0.0472 (3)	
C31	−0.2796 (2)	0.40101 (12)	0.01197 (10)	0.0333 (3)	
H31A	−0.3782	0.4129	0.0558	0.040*	
H31B	−0.2810	0.4607	−0.0053	0.040*	
C7	2.0845 (2)	0.78185 (12)	0.79254 (10)	0.0327 (3)	
C30	−0.0726 (2)	0.39840 (12)	0.05502 (10)	0.0337 (3)	
H30A	0.0267	0.3889	0.0119	0.040*	
H30B	−0.0698	0.3374	0.0707	0.040*	
O3	2.54720 (19)	0.71434 (9)	0.87183 (8)	0.0418 (3)	
C32	−0.3442 (2)	0.30171 (12)	−0.06881 (10)	0.0348 (4)	
H32A	−0.3433	0.2421	−0.0514	0.042*	
H32B	−0.2450	0.2897	−0.1124	0.042*	
C12	2.0006 (3)	0.87875 (12)	0.82186 (10)	0.0365 (4)	
H12	2.0747	0.9378	0.8653	0.044*	
N6	2.3818 (2)	0.85718 (11)	0.90254 (9)	0.0388 (3)	
N4	2.3705 (2)	0.68521 (11)	0.81288 (9)	0.0389 (3)	
C29	−0.0119 (2)	0.49672 (12)	0.13743 (10)	0.0339 (3)	
H29A	−0.1090	0.5053	0.1813	0.041*	
H29B	−0.0178	0.5580	0.1222	0.041*	
C10	1.7059 (2)	0.80337 (12)	0.72431 (10)	0.0311 (3)	
C34	−0.6142 (2)	0.20383 (12)	−0.19294 (10)	0.0357 (4)	
H34A	−0.5177	0.1933	−0.2374	0.043*	
H34B	−0.6067	0.1444	−0.1754	0.043*	
C8	1.9776 (2)	0.69561 (12)	0.72722 (10)	0.0323 (3)	
H8	2.0353	0.6295	0.7065	0.039*	
N22	0.7992 (2)	0.55954 (11)	0.38631 (9)	0.0413 (4)	
C9	1.7885 (2)	0.70490 (12)	0.69204 (10)	0.0314 (3)	
C24	0.5515 (2)	0.67201 (12)	0.36269 (10)	0.0331 (3)	
C36	−0.8799 (3)	0.10077 (13)	−0.31550 (11)	0.0409 (4)	
H36A	−0.7808	0.0904	−0.3588	0.049*	
H36B	−0.8732	0.0420	−0.2971	0.049*	
C18	1.2226 (2)	0.70293 (12)	0.53997 (10)	0.0337 (3)	
H18A	1.3308	0.6702	0.5025	0.040*	
H18B	1.2189	0.6737	0.5849	0.040*	
C20	0.8824 (2)	0.73138 (12)	0.46614 (10)	0.0313 (3)	
H20	0.8742	0.8051	0.4895	0.038*	
C5	2.2806 (2)	0.77262 (12)	0.83423 (10)	0.0329 (3)	
C21	0.7448 (2)	0.65460 (12)	0.40427 (10)	0.0309 (3)	
C11	1.8104 (3)	0.89102 (12)	0.78886 (10)	0.0354 (4)	
C37	−1.0859 (3)	0.09851 (13)	−0.35931 (10)	0.0387 (4)	
H37A	−1.1872	0.1026	−0.3181	0.046*	
H37B	−1.0970	0.1597	−0.3744	0.046*	
C17	1.2696 (3)	0.82027 (12)	0.58603 (11)	0.0380 (4)	
H17A	1.2454	0.8517	0.5428	0.046*	
H17B	1.1780	0.8511	0.6326	0.046*	
C19	1.0283 (2)	0.67627 (12)	0.48446 (10)	0.0314 (3)	
C16	1.4831 (3)	0.84709 (13)	0.62690 (11)	0.0380 (4)	
H16A	1.5766	0.8139	0.5817	0.046*	
H16B	1.5111	0.9236	0.6519	0.046*	
C2	2.5404 (3)	0.81666 (13)	0.92158 (11)	0.0374 (4)	
C13	1.6694 (3)	0.61197 (12)	0.62341 (11)	0.0401 (4)	
H13A	1.5376	0.6064	0.6431	0.060*	
H13B	1.7401	0.5484	0.6146	0.060*	
H13C	1.6528	0.6205	0.5678	0.060*	
C1	2.7090 (3)	0.86781 (15)	0.98960 (12)	0.0469 (4)	
H1A	2.7096	0.8402	1.0350	0.070*	
H1B	2.6934	0.9433	1.0165	0.070*	
H1C	2.8354	0.8537	0.9623	0.070*	
C14	1.7185 (3)	0.99591 (13)	0.82549 (12)	0.0475 (5)	
H14A	1.7798	1.0371	0.8854	0.071*	
H14B	1.5742	0.9857	0.8268	0.071*	
H14C	1.7419	1.0330	0.7880	0.071*	
O100	−1.4973 (6)	−0.0308 (2)	−0.5193 (2)	0.0642 (9)	0.5
H10A	−1.586 (8)	0.012 (4)	−0.504 (4)	0.096*	0.5
H10B	−1.503 (9)	−0.0960 (10)	−0.548 (3)	0.096*	0.5
O38	−1.1236 (2)	0.00445 (10)	−0.43770 (9)	0.0517 (4)	
H38	−1.238 (2)	0.006 (2)	−0.4585 (15)	0.077*	

### Atomic displacement parameters (Å^2^)

**Table:**

	*U*^11^	*U*^22^	*U*^33^	*U*^12^	*U*^13^	*U*^23^
N26	0.0317 (8)	0.0351 (7)	0.0377 (7)	0.0030 (6)	−0.0097 (6)	0.0109 (6)
O15	0.0290 (6)	0.0373 (6)	0.0372 (6)	0.0034 (5)	−0.0018 (5)	0.0159 (5)
O23	0.0362 (7)	0.0310 (6)	0.0464 (7)	0.0040 (5)	−0.0147 (5)	0.0066 (5)
C33	0.0279 (8)	0.0327 (8)	0.0349 (8)	0.0021 (6)	−0.0032 (6)	0.0067 (6)
C28	0.0285 (8)	0.0344 (8)	0.0335 (8)	0.0024 (6)	−0.0040 (6)	0.0114 (6)
C27	0.0296 (9)	0.0393 (9)	0.0353 (8)	0.0039 (7)	−0.0063 (7)	0.0109 (7)
C35	0.0291 (8)	0.0341 (8)	0.0362 (8)	0.0043 (7)	−0.0038 (7)	0.0055 (6)
O25	0.0417 (7)	0.0386 (6)	0.0514 (7)	0.0096 (6)	−0.0092 (6)	0.0104 (5)
C31	0.0280 (8)	0.0327 (8)	0.0331 (8)	0.0018 (6)	−0.0032 (6)	0.0084 (6)
C7	0.0304 (8)	0.0343 (8)	0.0336 (8)	0.0039 (6)	0.0003 (6)	0.0145 (6)
C30	0.0291 (8)	0.0341 (8)	0.0335 (8)	0.0019 (6)	−0.0024 (6)	0.0102 (6)
O3	0.0372 (7)	0.0394 (6)	0.0455 (7)	0.0085 (5)	−0.0034 (5)	0.0150 (5)
C32	0.0298 (9)	0.0353 (8)	0.0314 (8)	0.0017 (7)	−0.0030 (6)	0.0064 (6)
C12	0.0367 (9)	0.0308 (8)	0.0366 (8)	0.0010 (7)	−0.0048 (7)	0.0098 (6)
N6	0.0373 (8)	0.0369 (7)	0.0375 (7)	0.0054 (6)	−0.0039 (6)	0.0114 (6)
N4	0.0345 (8)	0.0379 (7)	0.0417 (7)	0.0047 (6)	−0.0034 (6)	0.0147 (6)
C29	0.0281 (8)	0.0343 (8)	0.0329 (8)	0.0011 (6)	−0.0047 (6)	0.0086 (6)
C10	0.0285 (8)	0.0327 (8)	0.0316 (7)	0.0034 (6)	−0.0004 (6)	0.0133 (6)
C34	0.0293 (8)	0.0344 (8)	0.0328 (8)	0.0055 (7)	−0.0035 (6)	0.0038 (6)
C8	0.0329 (9)	0.0300 (7)	0.0334 (8)	0.0051 (6)	0.0029 (6)	0.0120 (6)
N22	0.0348 (8)	0.0352 (7)	0.0421 (8)	0.0040 (6)	−0.0131 (6)	0.0067 (6)
C9	0.0326 (9)	0.0304 (7)	0.0302 (7)	0.0017 (6)	0.0009 (6)	0.0116 (6)
C24	0.0301 (8)	0.0370 (8)	0.0314 (7)	0.0006 (7)	−0.0029 (6)	0.0141 (6)
C36	0.0328 (9)	0.0357 (8)	0.0389 (9)	0.0042 (7)	−0.0078 (7)	0.0013 (7)
C18	0.0278 (8)	0.0345 (8)	0.0339 (8)	0.0029 (6)	−0.0032 (6)	0.0099 (6)
C20	0.0287 (8)	0.0297 (7)	0.0320 (7)	0.0015 (6)	−0.0006 (6)	0.0095 (6)
C5	0.0321 (9)	0.0341 (8)	0.0334 (8)	0.0039 (7)	0.0025 (6)	0.0147 (6)
C21	0.0277 (8)	0.0329 (8)	0.0294 (7)	0.0026 (6)	−0.0012 (6)	0.0106 (6)
C11	0.0355 (9)	0.0298 (7)	0.0373 (8)	0.0041 (7)	−0.0022 (7)	0.0110 (6)
C37	0.0308 (9)	0.0386 (9)	0.0355 (8)	0.0003 (7)	−0.0039 (7)	0.0049 (7)
C17	0.0330 (9)	0.0354 (8)	0.0414 (9)	0.0001 (7)	−0.0076 (7)	0.0134 (7)
C19	0.0292 (8)	0.0302 (7)	0.0302 (7)	0.0025 (6)	−0.0008 (6)	0.0082 (6)
C16	0.0351 (9)	0.0383 (8)	0.0413 (9)	−0.0027 (7)	−0.0082 (7)	0.0192 (7)
C2	0.0366 (9)	0.0378 (8)	0.0367 (8)	0.0060 (7)	0.0005 (7)	0.0143 (7)
C13	0.0367 (10)	0.0320 (8)	0.0429 (9)	0.0028 (7)	−0.0029 (7)	0.0076 (7)
C1	0.0397 (10)	0.0508 (10)	0.0451 (10)	0.0044 (8)	−0.0072 (8)	0.0164 (8)
C14	0.0488 (12)	0.0321 (8)	0.0512 (10)	0.0087 (8)	−0.0088 (9)	0.0085 (7)
O100	0.0418 (18)	0.0401 (18)	0.085 (3)	−0.0014 (17)	−0.0171 (18)	0.0032 (16)
O38	0.0355 (7)	0.0480 (7)	0.0479 (7)	−0.0014 (6)	−0.0147 (6)	−0.0013 (6)

### Geometric parameters (Å, º)

**Table:**

N26—H26	0.8800	C10—C9	1.406 (2)
N26—C27	1.4614 (19)	C10—C11	1.394 (2)
N26—C24	1.3352 (19)	C34—H34A	0.9900
O15—C10	1.3854 (18)	C34—H34B	0.9900
O15—C16	1.4356 (19)	C8—H8	0.9500
O23—N22	1.4056 (17)	C8—C9	1.387 (2)
O23—C19	1.3591 (18)	N22—C21	1.308 (2)
C33—H33A	0.9900	C9—C13	1.504 (2)
C33—H33B	0.9900	C24—C21	1.494 (2)
C33—C32	1.525 (2)	C36—H36A	0.9900
C33—C34	1.521 (2)	C36—H36B	0.9900
C28—H28A	0.9900	C36—C37	1.506 (2)
C28—H28B	0.9900	C18—H18A	0.9900
C28—C27	1.507 (2)	C18—H18B	0.9900
C28—C29	1.524 (2)	C18—C17	1.523 (2)
C27—H27A	0.9900	C18—C19	1.487 (2)
C27—H27B	0.9900	C20—H20	0.9500
C35—H35A	0.9900	C20—C21	1.412 (2)
C35—H35B	0.9900	C20—C19	1.349 (2)
C35—C34	1.520 (2)	C11—C14	1.510 (2)
C35—C36	1.522 (2)	C37—H37A	0.9900
O25—C24	1.2242 (19)	C37—H37B	0.9900
C31—H31A	0.9900	C37—O38	1.4329 (19)
C31—H31B	0.9900	C17—H17A	0.9900
C31—C30	1.521 (2)	C17—H17B	0.9900
C31—C32	1.520 (2)	C17—C16	1.510 (2)
C7—C12	1.391 (2)	C16—H16A	0.9900
C7—C8	1.394 (2)	C16—H16B	0.9900
C7—C5	1.473 (2)	C2—C1	1.481 (2)
C30—H30A	0.9900	C13—H13A	0.9800
C30—H30B	0.9900	C13—H13B	0.9800
C30—C29	1.521 (2)	C13—H13C	0.9800
O3—N4	1.4194 (18)	C1—H1A	0.9800
O3—C2	1.339 (2)	C1—H1B	0.9800
C32—H32A	0.9900	C1—H1C	0.9800
C32—H32B	0.9900	C14—H14A	0.9800
C12—H12	0.9500	C14—H14B	0.9800
C12—C11	1.392 (2)	C14—H14C	0.9800
N6—C5	1.387 (2)	O100—O100^i^	0.847 (5)
N6—C2	1.293 (2)	O100—H10A	0.834 (10)
N4—C5	1.304 (2)	O100—H10B	0.841 (10)
C29—H29A	0.9900	O38—H38	0.819 (10)
C29—H29B	0.9900		
			
C27—N26—H26	119.5	C8—C9—C10	118.41 (14)
C24—N26—H26	119.5	C8—C9—C13	121.52 (14)
C24—N26—C27	121.03 (13)	N26—C24—C21	116.22 (14)
C10—O15—C16	115.72 (13)	O25—C24—N26	123.45 (15)
C19—O23—N22	109.09 (11)	O25—C24—C21	120.32 (14)
H33A—C33—H33B	107.7	C35—C36—H36A	108.9
C32—C33—H33A	108.9	C35—C36—H36B	108.9
C32—C33—H33B	108.9	H36A—C36—H36B	107.7
C34—C33—H33A	108.9	C37—C36—C35	113.23 (14)
C34—C33—H33B	108.9	C37—C36—H36A	108.9
C34—C33—C32	113.38 (13)	C37—C36—H36B	108.9
H28A—C28—H28B	107.9	H18A—C18—H18B	107.8
C27—C28—H28A	109.1	C17—C18—H18A	109.1
C27—C28—H28B	109.1	C17—C18—H18B	109.1
C27—C28—C29	112.36 (13)	C19—C18—H18A	109.1
C29—C28—H28A	109.1	C19—C18—H18B	109.1
C29—C28—H28B	109.1	C19—C18—C17	112.46 (13)
N26—C27—C28	111.29 (13)	C21—C20—H20	127.9
N26—C27—H27A	109.4	C19—C20—H20	127.9
N26—C27—H27B	109.4	C19—C20—C21	104.27 (13)
C28—C27—H27A	109.4	N6—C5—C7	121.88 (14)
C28—C27—H27B	109.4	N4—C5—C7	123.71 (14)
H27A—C27—H27B	108.0	N4—C5—N6	114.37 (14)
H35A—C35—H35B	107.8	N22—C21—C24	120.05 (14)
C34—C35—H35A	109.1	N22—C21—C20	112.71 (14)
C34—C35—H35B	109.1	C20—C21—C24	127.21 (14)
C34—C35—C36	112.48 (13)	C12—C11—C10	118.13 (14)
C36—C35—H35A	109.1	C12—C11—C14	120.24 (15)
C36—C35—H35B	109.1	C10—C11—C14	121.58 (15)
H31A—C31—H31B	107.7	C36—C37—H37A	109.6
C30—C31—H31A	108.8	C36—C37—H37B	109.6
C30—C31—H31B	108.8	H37A—C37—H37B	108.1
C32—C31—H31A	108.8	O38—C37—C36	110.40 (13)
C32—C31—H31B	108.8	O38—C37—H37A	109.6
C32—C31—C30	113.69 (13)	O38—C37—H37B	109.6
C12—C7—C8	119.31 (15)	C18—C17—H17A	109.1
C12—C7—C5	118.79 (14)	C18—C17—H17B	109.1
C8—C7—C5	121.86 (14)	H17A—C17—H17B	107.8
C31—C30—H30A	108.9	C16—C17—C18	112.46 (14)
C31—C30—H30B	108.9	C16—C17—H17A	109.1
C31—C30—C29	113.32 (13)	C16—C17—H17B	109.1
H30A—C30—H30B	107.7	O23—C19—C18	115.49 (13)
C29—C30—H30A	108.9	C20—C19—O23	109.31 (13)
C29—C30—H30B	108.9	C20—C19—C18	135.17 (14)
C2—O3—N4	106.40 (12)	O15—C16—C17	108.21 (14)
C33—C32—H32A	108.8	O15—C16—H16A	110.1
C33—C32—H32B	108.8	O15—C16—H16B	110.1
C31—C32—C33	113.98 (13)	C17—C16—H16A	110.1
C31—C32—H32A	108.8	C17—C16—H16B	110.1
C31—C32—H32B	108.8	H16A—C16—H16B	108.4
H32A—C32—H32B	107.7	O3—C2—C1	117.70 (15)
C7—C12—H12	119.3	N6—C2—O3	113.45 (15)
C7—C12—C11	121.41 (15)	N6—C2—C1	128.85 (16)
C11—C12—H12	119.3	C9—C13—H13A	109.5
C2—N6—C5	102.68 (13)	C9—C13—H13B	109.5
C5—N4—O3	103.10 (12)	C9—C13—H13C	109.5
C28—C29—H29A	109.0	H13A—C13—H13B	109.5
C28—C29—H29B	109.0	H13A—C13—H13C	109.5
C30—C29—C28	112.80 (13)	H13B—C13—H13C	109.5
C30—C29—H29A	109.0	C2—C1—H1A	109.5
C30—C29—H29B	109.0	C2—C1—H1B	109.5
H29A—C29—H29B	107.8	C2—C1—H1C	109.5
O15—C10—C9	117.88 (13)	H1A—C1—H1B	109.5
O15—C10—C11	120.18 (14)	H1A—C1—H1C	109.5
C11—C10—C9	121.74 (14)	H1B—C1—H1C	109.5
C33—C34—H34A	108.7	C11—C14—H14A	109.5
C33—C34—H34B	108.7	C11—C14—H14B	109.5
C35—C34—C33	114.39 (13)	C11—C14—H14C	109.5
C35—C34—H34A	108.7	H14A—C14—H14B	109.5
C35—C34—H34B	108.7	H14A—C14—H14C	109.5
H34A—C34—H34B	107.6	H14B—C14—H14C	109.5
C7—C8—H8	119.5	O100^i^—O100—H10A	45 (5)
C9—C8—C7	120.98 (14)	O100^i^—O100—H10B	166 (4)
C9—C8—H8	119.5	H10A—O100—H10B	132 (6)
C21—N22—O23	104.61 (12)	C37—O38—H38	107.1 (18)
C10—C9—C13	120.03 (14)		
			
N26—C24—C21—N22	−9.9 (2)	C34—C33—C32—C31	−179.60 (14)
N26—C24—C21—C20	172.31 (15)	C34—C35—C36—C37	−179.10 (15)
O15—C10—C9—C8	−173.62 (14)	C8—C7—C12—C11	1.5 (3)
O15—C10—C9—C13	4.2 (2)	C8—C7—C5—N6	−176.35 (15)
O15—C10—C11—C12	174.16 (14)	C8—C7—C5—N4	1.3 (3)
O15—C10—C11—C14	−3.2 (3)	N22—O23—C19—C18	−177.78 (14)
O23—N22—C21—C24	−178.41 (14)	N22—O23—C19—C20	0.63 (18)
O23—N22—C21—C20	−0.32 (19)	C9—C10—C11—C12	−0.6 (2)
C27—N26—C24—O25	−4.6 (3)	C9—C10—C11—C14	−177.98 (16)
C27—N26—C24—C21	174.35 (14)	C24—N26—C27—C28	171.58 (14)
C27—C28—C29—C30	177.88 (14)	C36—C35—C34—C33	179.91 (15)
C35—C36—C37—O38	175.68 (15)	C18—C17—C16—O15	−64.32 (18)
O25—C24—C21—N22	169.10 (16)	C5—C7—C12—C11	−175.97 (15)
O25—C24—C21—C20	−8.7 (3)	C5—C7—C8—C9	176.58 (15)
C31—C30—C29—C28	−178.56 (14)	C5—N6—C2—O3	−0.4 (2)
C7—C12—C11—C10	−0.8 (3)	C5—N6—C2—C1	179.90 (18)
C7—C12—C11—C14	176.62 (17)	C21—C20—C19—O23	−0.78 (18)
C7—C8—C9—C10	−0.5 (2)	C21—C20—C19—C18	177.18 (18)
C7—C8—C9—C13	−178.27 (15)	C11—C10—C9—C8	1.2 (2)
C30—C31—C32—C33	−179.69 (14)	C11—C10—C9—C13	179.01 (15)
O3—N4—C5—C7	−178.08 (14)	C17—C18—C19—O23	176.76 (14)
O3—N4—C5—N6	−0.30 (19)	C17—C18—C19—C20	−1.1 (3)
C32—C33—C34—C35	177.44 (14)	C19—O23—N22—C21	−0.18 (18)
C32—C31—C30—C29	−178.21 (14)	C19—C18—C17—C16	−168.20 (14)
C12—C7—C8—C9	−0.8 (2)	C19—C20—C21—N22	0.69 (19)
C12—C7—C5—N6	1.1 (2)	C19—C20—C21—C24	178.63 (16)
C12—C7—C5—N4	178.67 (16)	C16—O15—C10—C9	−100.42 (16)
N4—O3—C2—N6	0.2 (2)	C16—O15—C10—C11	84.64 (18)
N4—O3—C2—C1	179.98 (15)	C2—O3—N4—C5	0.04 (17)
C29—C28—C27—N26	178.10 (14)	C2—N6—C5—C7	178.26 (15)
C10—O15—C16—C17	164.12 (13)	C2—N6—C5—N4	0.4 (2)

Symmetry code: (i) −*x*−3, −*y*, −*z*−1.

### Hydrogen-bond geometry (Å, º)

**Table:**

*D*—H···*A*	*D*—H	H···*A*	*D*···*A*	*D*—H···*A*
C28—H28*A*···O3^ii^	0.99	2.64	3.2567 (19)	120
C20—H20···O38^iii^	0.95	2.56	3.505 (2)	175
C13—H13*B*···O23^ii^	0.98	2.51	3.416 (2)	154
C1—H1*B*···N6	0.98	2.65	3.622 (2)	174
O100—H10*B*···O25^iv^	0.84 (1)	1.87 (1)	2.710 (3)	180 (6)
O38—H38···O100	0.82 (1)	1.90 (1)	2.695 (4)	164 (2)

Symmetry codes: (ii) −*x*+3, −*y*+1, −*z*+1; (iii) *x*+2, *y*+1, *z*+1; (iv) *x*−2, *y*−1, *z*−1.
